# Transposable Elements in Pluripotent Stem Cells and Human Disease

**DOI:** 10.3389/fgene.2022.902541

**Published:** 2022-06-02

**Authors:** Gang Ma, Isaac A. Babarinde, Xuemeng Zhou, Andrew P. Hutchins

**Affiliations:** Shenzhen Key Laboratory of Gene Regulation and Systems Biology, Department of Biology, School of Life Sciences, Southern University of Science and Technology, Shenzhen, China

**Keywords:** transposable element (TE), endogenous retrovirus (ERV), long terminal repeat (LTR), pluripotent stem cell (PSC), non-coding RNA (ncRNAs)

## Abstract

Transposable elements (TEs) are mobile genetic elements that can randomly integrate into other genomic sites. They have successfully replicated and now occupy around 40% of the total DNA sequence in humans. TEs in the genome have a complex relationship with the host cell, being both potentially deleterious and advantageous at the same time. Only a tiny minority of TEs are still capable of transposition, yet their fossilized sequence fragments are thought to be involved in various molecular processes, such as gene transcriptional activity, RNA stability and subcellular localization, and chromosomal architecture. TEs have also been implicated in biological processes, although it is often hard to reveal cause from correlation due to formidable technical issues in analyzing TEs. In this review, we compare and contrast two views of TE activity: one in the pluripotent state, where TEs are broadly beneficial, or at least mechanistically useful, and a second state in human disease, where TEs are uniformly considered harmful.

## Introduction

Transposable elements (TEs) are mobile genetic elements that are found in multiple copies in the genome. TEs were first discovered as mutable loci in 1944, in the study of the corn kernel and leaf color variegation in maize (McClintock, 1950). Barbara McClintock proposed the concepts of genetic loci termed activator and dissociation that could influence gene activity by changing their positions on chromosomes (Guffanti et al., 2014). TEs are usually grouped into two main classes based on transposition mechanism and structural features: the retrotransposons, which transpose by “copy and paste” through an RNA intermediate, and the DNA transposons, which change their positions by a “cut and paste” mechanism (Finnegan, 1989). Retrotransposons are further subdivided into long interspersed elements (LINEs), short interspersed elements (SINEs), and long terminal repeats (LTRs) which are endogenous retroviruses (ERVs). In the human genome, around 40% of the genome is composed of TEs (Hutchins and Pei, 2015), especially retrotransposons. The LINEs are the single most frequent TE, followed by the SINEs, which are a “parasite on a parasite” as they rely on LINE-encoded proteins for their transposition. The LTRs take up third place with DNA transposons and other TEs in last place (Hutchins and Pei, 2015).

The functionality of TEs has always been under some debate. Back in 1972, Sozumu Ohno termed TEs as “junk” DNA in the Brookhaven Symposium in Biology journal (Ohno, 1972). Indeed, there is some sympathy for this view even today, as the vast majority of TEs inside the genome are molecular fossils that have lost their original transposition function, especially in the human genome (Callinan and Batzer, 2006; Hellen and Brookfield, 2013). Meanwhile, even with the development of sophisticated sequencing technologies and genome-wide screens, most TE sequences remain uncharacterized due to difficulties in unambiguously identifying TEs from sequence reads. Thus, there is a persistent argument that TEs are functionally unimportant and are transcriptional or biological noise. Nevertheless, increasing research has suggested a role for at least some TEs in a wide range of biological processes, including genome evolution, gene organization, expression regulation, and numerous other aspects of cellular biology (Bourque et al., 2018). Consequently, there is an evolving view of TEs as both a potential source of genome innovation, and a potential danger to genome stability, and the development of disease (Payer and Burns, 2019). In this review, we will discuss two areas of TE action, in human pluripotent stem cells (hPSCs) and human disease.

### Features and Polymorphism of Transposable Elements

TE abundance varies from several copies up to several thousands of copies of the same element. Although any individual element of the same type is different, due to mutation and truncations, they nonetheless retain some homology. Indeed, even though a TE is inactive due to truncations or mutations, high copy number TEs with similar sequences can provide a sufficient template for recombination and genome rearrangement (Gray, 2000; Bourque et al., 2018). Thus, even as inactive fossils, TEs can still contribute to genome rearrangements. Duplicating TEs in the genome can produce insertion, deletions, chromosomal fusions, and even more complex chromosome rearrangements (Bourque et al., 2018). TE insertions are thus a potential major source of harmful mutations that can cause DNA double-strand breaks, gene dysfunction, gene recombination, gene expression dysregulation, and other types of mutations. TEs are thus a potent source of genetic polymorphisms.

Newly inserted mobile elements could lead to structural variants including deletions, insertions, duplications, and inversions, which may exist as polymorphisms within the population. Strikingly, there are more than 16,000 polymorphic TEs in the human genome, accounting for ∼24% of all known structural variants; many of these are common variants, with over 6,500 (36%) having a minor allele frequency >0.01 (Sudmant et al., 2015; Percharde et al., 2018). These insertions are generally located in hotspots with open chromatin. The alleles with novel TE insertions may differ from one another by poly-A tail length or nucleotide substitutions. These polymorphic elements reflect recent sequence insertions with few mutations. Furthermore, inserted TEs usually contain intrinsic functional sequences. Depending on the type of TE, insertional polymorphisms can include autonomous promoters, enhancers, and other regulatory sequences leading to heterochromatin formation, labeled secondary RNA or DNA structures, splicing regulators, and protein-coding sequences (Rishishwar et al., 2015; Bourque et al., 2018; Spirito et al., 2019). Therefore, it is critical to understand TE polymorphism, which directly affects the genetic diversity and the function of genes in the host genome.

### Molecular Functions and Co-Option of Protein-Coding Transposable Element Sequences

**FIGURE 1:**
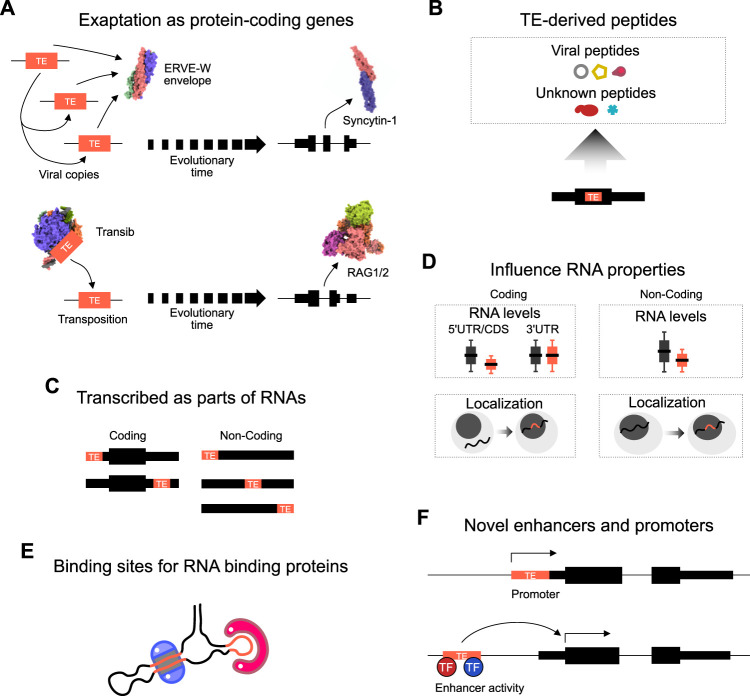
Mechanisms of TE activity. **(A)** Schematic of two examples of the exaptation of transposable element coding sequences as protein-coding genes. TEs randomly duplicate themselves in the genome, and eventually over evolutionary time one TE copy is exapted for biological function. A HERVW envelope protein became Syncytin, and a Transib became RAG1/2 in the immune system. Structures are from 1qbz (SIV gp41) (Yang et al., 1999), 5ha6 (Syncytin-1), 6pr5 (HzTransib) (Liu et al., 2019), 4wwx (RAG1/2) (Kim et al., 2015). **(B)** TEs inserted into coding frames can also give rise to TE-derived peptide fragments. Some match to known TE peptide sequences, for example, LINE ORFs, or ERV gag, pol, env proteins, yet other proteins have no match. **(C)** TEs can be transcribed as parts of coding or non-coding RNAs. In coding transcripts, most TEs are part of the 5′ or 3′ untranslated regions (UTR). In non-coding transcripts, TEs can be embedded anywhere inside the transcript, and non-coding transcripts often contain multiple different types of TE. **(D)** The presence of TEs inside an RNA sequence impacts properties of the transcript. For example, TEs tend to lead to reduced expression in non-coding transcripts, but only reduce coding RNAs when the TE is present in the 5′UTR or coding sequence (CDS). The presence of a TE in both coding and non-coding transcripts uniformly leads to increased retention of the transcript in the nucleus. **(E)** TEs can form binding sites for the recognition of and binding by RNA binding proteins. RNAs fold up to form hairpins and complex structures which are recognized by sequence or structure-specific RBPs. **(F)** TEs, when inserted into the genome can lead to the evolution of novel enhancers or promoters. TEs contain promoters to promote their expression (and so enhance their ability to colonize the genome). However, TEs can also form novel promoter transcription start sites. TEs also contain transcription factor binding sites that can recruit endogenous transcription factors (TFs) to activate nearby gene expression.

Over the past few decades, scientists have mainly focused on several areas in the study of TEs, particularly the annotation, classification, and evolution of TEs, and less emphasis has been paid to their functions. However, recent studies have increasingly shown that TEs play a vital role in a wide range of biological processes (Guffanti et al., 2014; Saleh et al., 2019). TEs can regulate gene transcription (Anwar et al., 2017), RNA subcellular distribution, RNA half-life, transcript abundance and splicing, and DNA methylation (Bourque et al., 2018). Strikingly, TE-encoded proteins have been co-opted for genuine biological function. For example, RAG1 and RAG2, which are essential for mediating sequence-specific DNA recognition in immunoglobulin and T-cell-receptor genes assembly, are derived from a transposase (Agrawal et al., 1998; Zhang et al., 2019) (Figure 1A). Syncytin-1 is another co-opted protein, in this case, derived from the viral envelope of a HERV-W (Figure 1A). Syncytin-1 has been proposed to have critical roles in normal human placental morphogenesis (Mi et al., 2000). Syncytin-1 is also high in the brains of schizophrenia patients, this correlates with the expression of the inflammation marker CRP (Wang et al., 2018). In addition to these examples, peptides derived from TE sequences have been detected in several cell types (Grow et al., 2015; Li et al., 2015; Babarinde et al., 2021) (Figure 1B), although their function (if any) remains unclear.

In addition to the direct co-option of protein-coding TE sequences, TE sequence fragments also play subtle and complex roles in other cellular processes. TE sequences can be transcribed and can influence RNA activity when TE-derived sequence fragments are embedded in RNA sequences (Thornburg et al., 2006) (Figure 1C). Indeed, noncoding RNAs are rich in TE-derived sequences (Kelley and Rinn, 2012; Kapusta et al., 2013). Many HERV- and LINE-derived long noncoding transcripts are enriched in stem cells and numerous disease models (Thomas et al., 2017; Babarinde et al., 2021). There is also evidence that the TE sequences inside RNAs influence RNA properties (Figure 1D). For example, the presence of TEs in both coding and non-coding transcripts tends to result in lower expression levels, and TEs can lead to retention of the RNA inside the nucleus (Faulkner et al., 2009; Carlevaro-Fita et al., 2019; Babarinde et al., 2021). The mechanism is unclear, but TEs form binding sites that are recognized by RNA binding proteins (Figure 1E) (Van Nostrand et al., 2020). On the other hand, epigenomic and transcriptomic studies have revealed that TE sequences contribute a significant fraction of species- and tissue-specific regulatory elements (Kunarso et al., 2010; Trizzino et al., 2017; Pehrsson et al., 2019). TEs often contain a promoter and novel insertions can thus generate new promoter regions of existing genes or generate new non-coding RNAs (Figure 1F). Indeed, many transcripts start inside TE sequences, or TEs can provide alternative promoters or antisense transcripts (Faulkner et al., 2009). TEs can alter gene expression patterns by contributing cell-specific transcription factor binding sites (Thornburg et al., 2006), which function as enhancers to drive gene expression (Figure 1F), for example, several MER41 elements carry out enhancer functions of interferon response genes (Chuong et al., 2016). Overall, TEs can be utilized by the cell in several ways for legitimate biological functions.

## Transposable Elements in Stem Cells

### Activity of Fossil Transposable Elements in Pluripotent Stem Cells

TEs have been reported to be expressed in a highly tissue-specific manner (Ecco et al., 2016; Ashapkin et al., 2019; He et al., 2021). Although TEs are expressed in numerous cell and tissue types (Ecco et al., 2016; Ashapkin et al., 2019), they are especially active in early embryonic development (Goke et al., 2015; Barakat et al., 2018; Percharde et al., 2018; Wang et al., 2020). During early embryonic development, specific types of TE are expressed in a stage-specific manner (Goke et al., 2015; Wang et al., 2020). Interestingly, TE activity in hPSCs coincides with the relaxed genome structure, global hypomethylation, and general genome activation (Zamudio and Bourc’his, 2010; Guo et al., 2014).

To explain the high activity of TEs in early embryo cells and hPSCs, two hypotheses have been proposed. The first suggests that the relatively high activity of the TEs in hPSCs is a consequence of the strategies used by TEs to duplicate themselves across generations (Gerdes et al., 2016). If a TE can duplicate itself in the embryonic or germline cells it will have a higher chance to pass to the next generation, hence TEs often contain embryonic-specific transcription factor binding sites to promote their expression. The second hypothesis takes the opposite approach: that TE activity in hPSCs is an evolutionary innovation of the host cell, and instead of excessively silencing TEs, the host cells intentionally permit the activity of TEs in hPSCs (Brennecke et al., 2008; Sidorenko et al., 2017). The decision of the host cells to permit TE activity might have conferred at least two advantages. First, TE sequence diversity is a potential resource for evolutionary innovation (Heng et al., 2010; Rodriguez-Terrones and Torres-Padilla, 2018). Second, it has been proposed that the host cells transiently relax TE repression during embryogenesis as a way to recognize active TEs and so later activate efficient TE repressive mechanisms during development (Zamudio and Bourc’his, 2010). Indeed, this latter mechanism is employed in the germline cells where TEs are transiently activated during genome reprogramming and are silenced by the PIWI Interacting RNA (piRNA) pathway (Castaneda et al., 2011; Yang and Wang, 2016; Hurst and Magiorkinis, 2017). This class of hypotheses suggests that host cells are the main determinant of TE activities, which makes sense considering the vast majority of TEs are mutated and no longer functional.

Only a handful of TEs (<0.05%) are still capable of transposition in the human genome (Hormozdiari et al., 2011), the majority are molecular fossils. Hence, TE activity doesn’t just mark the pluripotent state; they have also been proposed to perform functions in normal embryonic genome activation and development (Mi et al., 2000; Haig, 2016; Izsvak et al., 2016). Some evidence for this comes from the highly stage-specific expression of TEs, for example, SINE Alu is active at the 4-8 cell stage in human (Gerdes et al., 2016), and LINE-1 is highly expressed in hPSCs (Wang et al., 2020). While LTR5-HERVK is mainly activated from the 8-cell stage to blastocyst (Goke et al., 2015). There is also some evidence that the TE sequences themselves are involved in embryogenesis, for example, knocking down HERVHs in hPSCs leads to differentiation (Lu et al., 2014). Nonetheless, there remains a large number of TE sequence fragments expressed in the early embryo and in hPSCs that have no assigned function.

### Transposable Elements Are Components of Embryonic Regulatory Networks

TE activity is tightly regulated in biological systems. In somatic cells, the regulation of TE activities is controlled by DNA methylation (Zamudio and Bourc’his, 2010; Guo et al., 2014). However, in early embryogenesis DNA is demethylated and in naïve hPSCs, the majority of human TEs are hypomethylated (Gkountela et al., 2015). Instead of repression by DNA methylation, TEs are suppressed by a range of mechanisms, mainly histone modifications, such as Histone 3 lysine 9 trimethylation (H3K9me3), mediated by KRAB-ZNFs through their cofactor TRIM28 which recruits the H3K9me3 methyltransferase SETDB1 (Castro-Diaz et al., 2014; Turelli et al., 2014; Pontis et al., 2019). Specifically, depletion of TRIM28 led to the removal of the repressive chromatin marks, thereby activating ERVs (Rowe et al., 2013; Turelli et al., 2014). Indeed, ZNF93 and TRIM28 mediate the deposition of H3K9me3 on a subset of LINE-1 elements to repress their transcription (Castro-Diaz et al., 2014), while the transcription factor YY1 is involved in indirectly silencing younger LINE-1 subfamilies by mediating DNA methylation in hPSCs (Sanchez-Luque et al., 2019). However, the regulation of TEs is more complex than just repression, as TEs in mouse PSCs harbor a wide range of histone patterns, including histone marks for active transcription and enhancers (He et al., 2019; Zhou et al., 2021). Taken together, TEs are regulated by a large array of biological processes such as DNA methylation, histone modification, RNA degradation, and translational control.

**FIGURE 2:**
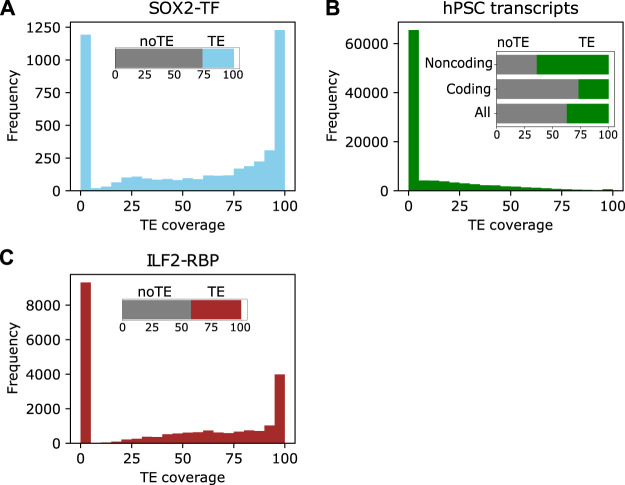
TEs are incorporated into different elements in stem cells. **(A)** The distribution of TE coverage in SOX2 binding sites in human naïve PSCs. The data were retrieved from (Huang et al., 2021). **(B)** The distribution of TEs in the hPSC transcriptome assembly. The insert shows the distribution of TE-sequences in all, coding and noncoding transcripts. **(C)**. The distribution of TEs in ILF2 RNA binding sites. The insert is the total classification based on any TE overlap.

TEs play important roles in the regulatory networks of ESC (Barakat et al., 2018; Wang et al., 2020; Bakoulis et al., 2022). They contain binding sites of key hPSC-specific regulatory factors, such as POU5F1, SOX2, and NANOG (Wang et al., 2020). For example, more than 70% of the SOX2 binding sites in hPSCs overlapped a TE (Figure 2A). The relationship between hPSC-specific factors and TEs is also mechanistic, as disruption of naïve pluripotent stem cells transcription factor LBP9 downregulated the transcription of HERVH-derived transcripts and compromised the self-renewal of hPSCs (Wang et al., 2014). Importantly, TEs have been incorporated into normal regulatory networks to enable species-specific innovations (Heng et al., 2010; Rodriguez-Terrones and Torres-Padilla, 2018). For instance, a comparison of the genome-wide binding locations of two pluripotency transcription factors POU5F1 and NANOG in human and mouse PSCs found that the binding locations of POU5F1 and NANOG were highly divergent between the two species, and around a quarter of the transcription factor binding sites were contained inside TE sequences (Kunarso et al., 2010). Ultimately, the study suggested that the activity of TEs moves transcription factor binding sites throughout the genome over evolutionary time. In this way, transcription factors can regulate the same genes in the same cell type, but the binding sites themselves need not be well conserved.

The transcription of TEs in human pluripotent stem cells (hPSCs) has been extensively investigated. Several studies have reported various proportions of TEs in human transcripts, it is widely agreed that TEs contribute more to lncRNAs than protein-coding transcripts, suggesting they are a major component of lncRNAs (Babarinde et al., 2021). For example, 64% of lncRNAs in hPSCs contain a TE-derived sequence, compared to 27% of coding transcripts (Babarinde et al., 2021). Interestingly, TE-sequence-containing transcripts also tended to contain varying proportions of unique sequences (Figure 2B), demonstrating that TEs have been incorporated into different hPSC transcripts to various degrees (Babarinde et al., 2021). Additionally, TE-containing hPSC transcripts tend to be less stable, more localized to the nucleus, and with less coding ability (Babarinde et al., 2021). One well-studied example of a TE-derived lncRNA in human ESCs is HERVH transcripts. Functionally, the disruption of HERVH and HERVH-derived transcripts negatively affects the self-renewal of hPSCs by reducing the recruitment of pluripotent transcription factors to particular binding sites (Lu et al., 2014; Wang et al., 2014). These studies highlight the roles of TE-derived transcripts in hPSCs.

### Transposable Elements Are Targets of RNA-Binding Proteins in Human Pluripotent Stem Cells

Transcript stability and subcellular localizations are largely controlled by RNA binding proteins (Kelley et al., 2014). Mechanistically, TEs may lead to changes in RNA activities through binding to RNA binding proteins (RBPs). By mapping the RNA binding sites of 51 human proteins, Kelley et al. (2014) found that the RNA binding proteins (RBPs) were differentially bound to specific TEs. A large-scale genome-wide study also found RBPs bound to antisense sequences of LINEs and Alu SINE TEs (Van Nostrand et al., 2020). This suggests a possible mechanism for TEs to alter RNA properties, such as transcript abundance and splicing. Indeed, the TEs themselves may form binding sites for RBPs, for example in how STAUFEN binds to Alu SINE sequences (Gong and Maquat, 2011).

A similar pattern is seen in hPSCs, and TE-sequences in RNAs are bound by RBPs. Analysis of the RBPs DDX6, ILF2, FUS, and DCP1B in hPSCs revealed that TE-containing transcripts have unique RBP interaction (Babarinde et al., 2021). For example, 58% of the RNA-binding sites for ILF2 identified are derived from TEs (Figure 2C). Functionally, DDX6 which has widespread binding sites with no TE preference in hPSC transcripts (Babarinde et al., 2021) is associated with cell plasticity and parental RNA decay for cellular reprogramming to pluripotency (Kami et al., 2018; Di Stefano et al., 2019). Despite limited studies reporting RBP interactions with TEs or TE-derived transcripts in hPSCs, the analyses of RBPs in other cell types (Kelley et al., 2014; Attig et al., 2018; Van Nostrand et al., 2020) as well as the abundance and properties of TEs incorporated into transcripts, point to the importance of the interplay between RBPs and TEs. TEs inside RNAs, and especially in lncRNAs, may form binding platforms or regulatory domains for RBPs to bind to and execute biological functions (Johnson and Guigo, 2014).

## Transposable Elements in Disease

The involvement of TEs in harmful mutations, gene dysfunction, DNA double-strand breaks, gene recombination, gene expression dysregulation, and other types of mutations implies that TEs might contribute to human disease. There is growing evidence of a link between TE sequence fragments, PSCs, and cancer. TEs can act as oncogene-specific enhancers, promoters, and exons for pluripotency-specific genes, this drives their expression and converts them into oncogenes (Jang et al., 2019). This builds on the observations that pluripotent genes are oncogenic (Leis et al., 2012; Muller et al., 2016), and PSCs themselves have tumorigenic potential. However, the TE-gene fusions seen in cancer were not observed in normal hPSCs (Zapatka et al., 2020; Babarinde et al., 2021). This observation suggests that some TEs are either normal or tolerated by the cell, whilst other TEs are specifically associated with disease.

TE transposition activity has been suggested to contribute to human genetic diseases, primarily through the transposition of LINE-1, SINE Alu, and SVA (SINE-VNTR-Alu) TEs (Cordaux and Batzer, 2009). Strikingly, nearly 100 retrotransposition events caused by polymorphic LINE-1 have been implicated in human diseases (Hancks and Kazazian, 2012). Non-functional mutated TE sequences expressed inside RNAs have also been implicated in several conditions, including psychiatric disorders (Guffanti et al., 2014), neurofibromatosis (Payer and Burns, 2019), cancer (Chenais, 2013; Rodriguez-Martin et al., 2016) and aging (De Cecco et al., 2013b). However, until recently, there were surprisingly few concrete examples of the impact of TEs on human disease (Nakamura et al., 2015; Rodriguez-Martin et al., 2016), and the association with disease often remains correlative. Much of the challenge in studying TE-associated human diseases stems from the difficulty in accurately sequencing the locations of TEs inside an individual’s genome, coupled with difficulties in establishing causation between novel insertions and disease. Table 1 shows example TEs that have been implicated in selected diseases.

**TABLE 1:** Transposable elements implicated in human disease.

Condition type	TEs	Mechanism	References
Cancer			
Colon cancer	LINE-1	LINE-1 promoter hypomethylation	(Ogino et al., 2008; Cruickshanks and Tufarelli, 2009; Weber et al., 2010)
Esophageal squamous cell carcinoma	LINE-1	LINE-1 promoter hypomethylation	Iwagami et al. (2013)
Breast cancer	LINE-1	LINE-1 promoter hypomethylation	(Cruickshanks and Tufarelli, 2009; van Hoesel et al., 2012)
Hepatocellular carcinomas	LINE-1	LINE-1 promoter hypomethylation	Harada et al. (2015)
Ovarian cancer	LINE-1	LINE-1 promoter hypomethylation	Pattamadilok et al. (2008)
Chronic myeloid leukemia	LINE-1	LINE-1 promoter hypomethylation	(Roman-Gomez et al., 2005; Weber et al., 2010)
Bladder tumors	LINE-1	LINE-1 promoter hypomethylation	Wolff et al. (2010)
Colorectal cancer	LINE-1	LINE-1 promoter hypomethylation	Hur et al. (2014)
Colon cancers	LINE-1	Forms dsRNA and suppresses *TFPI2*	Cruickshanks et al. (2013)
Colon cancer	LINE-1 ORF1p	LINE-1 ORF1p overexpression	Rodic et al. (2014)
Ovarian cancers	LINE-1 ORF1p	LINE-1 ORF1p overexpression	Rodic et al. (2014)
Lung cancers	LINE-1 ORF1p	LINE-1 ORF1p overexpression	Ardeljan et al. (2017)
Colon cancer	LINE-1	LINE-1 insertion into tumor suppressor APC	(Miki et al., 1992; Scott et al., 2016)
Colorectal cancer	LINE-1	Insertion causes gene mutation	(Lee et al., 2012; Solyom et al., 2012)
NSCLCs	LINE-1	Insertion causes gene mutation	(Iskow et al., 2010; Tubio et al., 2014)
Head and neck cancers	LINE-1	Insertion causes gene mutation	(Helman et al., 2014; Tubio et al., 2014)
Ovarian cancers	LINE-1	Insertion causes gene mutation	(Lee et al., 2012; Tubio et al., 2014; Tang et al., 2017)
Gastric cancer	LINE-1	LINE-1 hypomethylation	Shigaki et al. (2013)
Ovarian cancer	HERV-K	Increased expression	Rycaj et al. (2015)
Melanoma	HERV-K	Increased expression	(Schiavetti et al., 2002; Singh et al., 2020)
Pancreatic cancer	HERV-K	Increased expression	Li et al. (2017)
**Psychiatric disorders, neurofibromatosis, Alzheimer’s disease**			
Multiple sclerosis	HERV-W	LINE-1 expression	Perron et al. (1993)
Aicardi-goutières syndrome	LINE-1	Re-activates LINE-1	Thomas et al. (2017)
Rett syndrome	LINE-1 ORF2	MECP2 loss of function increases susceptibility to LINE-1 insertions	Muotri et al. (2010)
Systemic lupus erythematosus	HERV	Increased expression correlates with SLE	Wu et al. (2015)
Sporadic amyotrophic lateral sclerosis	HERV-K	Increased expression correlates with SALS	Li et al. (2015)
Autism spectrum disorders	LINE-1	An increase in LINE-1 expression correlates with autism	(Shpyleva et al., 2018; Tangsuwansri et al., 2018)
Amyotrophic lateral sclerosis	HERV-K	Aberrant expression	Curzio et al. (2020)
Multiple sclerosis	HERV-W	Increased expression	(Perron et al., 1997; Antony et al., 2007)
**Immune system**			
Fibromyalgia	HERVs	Increased expression correlates with fibromyalgia	Ovejero et al. (2020)
Autoimmunity	HERV envelope	Expression triggers both innate and adaptive immunity	Grandi and Tramontano, (2018)
**Aging**			
Age-associated inflammation	LINE-1	Derepresses LINE-1 and activates a type I interferon (IFN-I) response	De Cecco et al. (2019)
Senescence	Alu, SVA, and L1	More accessible for Alu, SVA, and L1 transcription	De Cecco et al. (2013a)
Aging	LINE-1	SIRT6 fails to repress LINE-1 activity	Van Meter et al. (2014)

### Transposable Elements in Cancer

LINE-1 elements are actively mobilized in cancer; however, untangling if this is a cause or consequence of tumorigenesis has been challenging. The first identified example of a LINE-1 disrupting a tumor suppressor gene was recognized in 1992 in a patient with colorectal cancer (Miki et al., 1992). It was found that a LINE-1 was inserted into the *APC* tumor suppressor gene. The insertion included the 3′ part of a LINE-1 and around 180 base pairs of polyadenylated sequence (Miki et al., 1992). This was the first report of the disruption of a tumor suppressor gene caused by the somatic insertion of a mobile genetic element. Another independent study also found a novel somatic LINE-1 insertion in colorectal cancer which disrupted the *APC* gene (Scott et al., 2016). Moreover, using LINE-1-targeted sequencing in 16 colorectal tumors, Szilvia confirmed tumorigenesis-related genes were mutagenized by specific *de novo* LINE-1 insertions (Solyom et al., 2012). The study validated 69/107 tumor-specific insertions including 35 instances in which both 5′ and 3′ junctions were retrieved. This agrees with the study of cancer genomes from 244 patients, in which 53% of the patients had somatic retrotranspositions, including 24% 3′ transductions (Tubio et al., 2014). By utilizing single-nucleotide resolution analysis, Lee et al. (2012) reported LINE-1 in genes that are commonly mutated in cancer. Identifying somatic TE insertions is experimentally challenging and a series of “Transposon-seq” methods have been developed that attempt to accurately place somatic TE insertions (Iskow et al., 2010). Employing a Transposon-seq method to interrogate 767 tumor samples with hybrid-capture exome data discovered 35 novel somatic transpositions, including one in the *PTEN* tumor suppressor gene (Helman et al., 2014). Also, LINE-1 ORF1p overexpression is observed in many human tumors (Burns, 2017). Indeed, LINE-1 expression has been positively identified as a biomarker in several cancers (Table 1).

Deregulation of TEs is a hallmark of many kinds of cancer (Jang et al., 2019; Zapatka et al., 2020). Genome-wide DNA hypomethylation is harmful to genomic stability and is often onbserved in cancer, particularly colorectal carcinogenesis (Jones and Gonzalgo, 1997; Lengauer et al., 1997; Breivik and Gaudernack, 1999; Ogino et al., 2008). In 2010, Wolff et al. (2010) for the first time demonstrated that the LINE-1 promoter hypomethylation directly causes dysregulation of endogenous gene expression. They reported that hypomethylation of the LINE-1 promoter activated an alternate transcript of the *MET* oncogene in bladder tumors. Recent studies have implicated LINE-1 promoter hypomethylation in various cancers by enabling full-length LINE-1 mRNA translation from the LINE-1 mobilization machinery (Shukla et al., 2013; Burns, 2017; Zhang et al., 2020). Indeed, LINE-1 promoter hypomethylation, LINE-1 ORF1p protein expression, and somatic polymorphic LINE-1 retrotransposition have been linked to lung, colon, pancreatic, ovarian, and breast cancers (Lee et al., 2012; van Hoesel et al., 2012; Rodic et al., 2014; Ardeljan et al., 2017). Further, LINE-1 promoter hypomethylation leading to sense/antisense transcription is a marker for the progression of chronic myeloid leukemia (Roman-Gomez et al., 2005). The LINE-1 antisense promoter can also affect adjacent genomic states by generating chimeric RNAs which can interrupt transcription. Novel LINE-1 chimeric transcripts have been observed in breast cancer cell lines and colon cancer cells (Cruickshanks and Tufarelli, 2009). These studies demonstrate that hypomethylation of LINE-1s plays a role not only in human diseases but also in disease predisposition (Wolff et al., 2010). Consistently, LINE-1 hypomethylation caused by the inhibition of DNMTs in colon carcinoma cells or myeloid leukemia cells induces the expression of an irregular fusion transcript between an intronic LINE-1 element and the proto-oncogene *MET* (c-Met) (Weber et al., 2010). In addition, an analysis of 77 colorectal cancer patients showed that hypomethylation of LINE-1 led to the activation of proto-oncogenes in human colorectal cancer metastasis (Hur et al., 2014). Also, genome-wide DNA hypomethylation levels revealed by LINE-1 hypomethylation demonstrated that LINE-1 methylation levels can be used as a biomarker for identifying hepatocellular carcinoma patients who will experience poor clinical outcomes (Pattamadilok et al., 2008; Harada et al., 2015). In addition, LINE-1 hypomethylation has been reported to be associated with poor survival in more than 200 cases of gastric cancer and esophageal squamous cell carcinoma (Iwagami et al., 2013; Shigaki et al., 2013), suggesting its potential as a prognostic biomarker. Further, the study applying pyrosequencing in two independent cohorts of 643 colon cancer patients found that LINE-1 hypomethylation is associated with shorter survival (Ogino et al., 2008). Taken together, LINE-1 hypomethylation leading to aberrant transcription is associated with various cancer types.

In addition to LINE-1, the expression of ERVs has also been reported in cancer (Zapatka et al., 2020), including translated peptides and fragments of viral proteins. Transcripts derived from ERVs have been observed in many cancer types, including particularly ovarian cancer (Rycaj et al., 2015), melanoma (Schiavetti et al., 2002), pancreatic cancer (Li et al., 2017), breast cancer (Wang-Johanning et al., 2008), and prostate adenocarcinoma (Wang-Johanning et al., 2003). However, the mechanistic roles of the ERV-containing transcripts and peptides has not been well explored. HERV-K is one of the most well-studied subfamilies of ERVs. For instance, downregulation of HERV-K decreased cell proliferation and tumor growth in pancreatic cancer (Li et al., 2017), suggesting a causal link between ERVs and tumor growth. Furthermore, the experimental depletion of HERV-K Rec in melanoma led to a lower level of melanocyte-inducing transcription factor (MITF), which may impact the transition from proliferative to invasive stages of melanoma (Singh et al., 2020). Targeting ERV sequences for downregulation has also been shown to have the potential in decreasing cancer proliferation in pancreatic cancers and melanoma (Li et al., 2017). These studies suggest that ERVs are not just markers for cancers, but they may also directly contribute to cancer progression, although the mechanisms behind these processes are not clear.

### Transposable Elements in Inflammation and Neuroderegulation

TEs have also been shown to be a key player in immune regulation, such as cancer immune and autoimmune diseases (Ovejero et al., 2020). For example, LINE-1 can manipulate the immune system and immune microenvironment in many ways (Zhang et al., 2020). The analysis of the transcriptional activity of 1789 pathways in 112 TCGA cancer samples revealed that 49 of 176 immune pathways were significantly negatively correlated with LINE-1 expression (Zhang et al., 2020). Jung et al. (2018) reported that cancer immunity may contribute to genome stability by suppressing LINE-1 retrotransposition in gastrointestinal cancers. Furthermore, the LINE-1 methylation level was significantly associated with the peritumoral lymphocytic reaction in esophageal cancer (Zhang et al., 2020). Besides the involvement in immune alteration of tumors, LINE-1 also plays an important role in other inflammation-related diseases. For example, intragenic LINE-1s can also act as *cis*-regulatory elements to mediate the activation of the autoimmune gene expression in Fanconi anemia and Aicardi-Goutières syndrome (Wanichnopparat et al., 2013). Also, accumulation of LINE-1 in *TREX1*-deficient type I interferon apoptosis leads to autoimmune neuroinflammation disease (Thomas et al., 2017). In human neurodevelopmental diseases model, *MECP2* mutations can influence the frequency of LINE-1 retrotransposition (Muotri et al., 2010). Moreover, LINE-1 expression was significantly elevated in Autism (Shpyleva et al., 2018). Apart from LINE-1, ERVs have also been reported capable of initiating an immune response in disease models. One example is Syncytin-1, which is normally expressed in the placenta but has been reported to be upregulated in multiple sclerosis (MS) lesions (Antony et al., 2011). The abnormal expression was proposed to lead to proinflammatory cytokine release, oxidative damage, and eventually oligodendrocyte death. Another study has shown that HERVs of the H, K, and W subfamilies are overexpressed in the immune cells of fibromyalgia patients (Ovejero et al., 2020). Interestingly, patients infected with severe acute respiratory syndrome coronavirus 2 (SARS-CoV-2), responsible for the COVID-19 pandemic, showed transcriptional upregulation of HERV-17 in T lymphocytes and leukocytes, which correlated with disease severity (Balestrieri et al., 2021; Garcia-Montojo & Nath, 2021).

Multiple studies have also implicated TEs in nervous system diseases such as neuropathy, psychiatric disorders, neurofibromatosis, and neurodegeneration. There is a particular interest in TE-derived peptides that induce an inflammatory response. For HERVK/Ws both their sequences and protein products have been implicated in the development of amyotrophic lateral sclerosis (ALS) and other neurological diseases (Arru et al., 2018). In fact, the inhibition of HERV-K expression with anti-viral therapy decelerated ALS progression (Arru et al., 2018). Interestingly, one study identified a conotoxin-like protein that was produced from an ERVK sequence as a potential factor in ALS neuropathology (Curzio et al., 2020). The conotoxin-like protein could induce NF-kB pro-inflammatory signaling, and its presence was linked with regions of damage in ALS samples (Curzio et al., 2020). Other studies have also documented various ways in which ERVs potentially contribute to multiple sclerosis (Perron et al., 1997; Antony et al., 2007). Moreover, the inhibition of LTR methylation may activate HERV-E transcription in systemic lupus erythematosus (Li et al., 2015; Wu et al., 2015), and elevated HERV-K expression in patients may contribute to neurodegeneration (Li et al., 2015). Indeed, the expression and DNA methylation patterns of ERV and LINE-1 are often disrupted in patients with autism, schizophrenia, and mood disorders (Misiak et al., 2019). These studies highlight various examples of the association between TEs and inflammation and neurodegeneration.

## Conclusion

TEs are active in different cell types and developmental stages, they are hyperactive in hPSCs where they function in transcriptional regulation, transcript processing such as splicing, RNA stability, and translational processes. Intriguingly, TEs are broadly positive in early embryogenesis, contributing to gene regulation pathways, and acting as a substrate for evolutionary innovation. This is in contrast to the role of TEs in somatic tissues, which tend to be more negative, being associated with the development of human diseases. TEs and their derived peptides or sequence fragments have complex roles in the cell. However, many of these roles remain unclear. Considering the vast number of TEs in the human genome it has, and remains, challenging to study them. However, there is likely much critical information that remains to be discovered concerning both the advantageous and deleterious functions of TEs in both embryogenesis and somatic cells.
